# Supplementary material for: BEACON: predicting side effects and therapeutics outcomes to drugs by Bridging knowlEdge grAph with CONtextual language model
Source: bioRxiv. 2026 Jan 30:2026.01.29.702277. Preprint. [Version 1] doi: 10.64898/2026.01.29.702277 (PMC12874035; doi:10.64898/2026.01.29.702277)
Supplement: 1 [file NIHPP2026.01.29.702277V1-supplement-1.pdf]

bioRxiv preprint doi: https://doi.org/10.64898/2026.01.29.702277; this version posted January 30, 2026. The copyright holder for this preprint (which was not certified by peer review) is the author/funder, who has granted bioRxiv a license to display the preprint in perpetuity. It is made available under aCC-BY 4.0 International license.

Supplementary material

Supplementary Tables

bioRxiv preprint doi: https://doi.org/10.64898/2026.01.29.702277; this version posted January 30, 2026. The copyright holder for this preprint (which was not certified by peer review) is the author/funder, who has granted bioRxiv a license to display the preprint in perpetuity. It is made available under aCC-BY 4.0 International license.

Supplementary Table 1. KG features are critical for model performance. On limited KG, BEACON still achieves robust performance compared to CNN and RNN.

Method	Drug features injected from biomedical Graph	Architecture	AUROC	AUPRC	Precision@50
BEACON (ablated KG with biological process alone)	pathway, targets	GPT framework	**0.910±0.005**	**0.835±0.003**	**0.829±0.003**
CNN (ablated KG with biological process alone)	pathway, targets	Convolutional Networks	0.762±0.002	0.601±0.005	0.702±0.004
RNN (ablated KG with biological process alone)	pathway, targets	Recurrent Neural Networks	0.574±0.001	0.395±0.004	0.660±0.003
BEACON (ablated KG with Pharmacological properties alone)	ATC, category	GPT framework	**0.938±0.002**	**0.873±0.003**	**0.852±0.006**
CNN (ablated KG with Pharmacological properties alone)	ATC, category	Convolutional Networks	0.815±0.003	0.683±0.004	0.734±0.002
RNN (ablated KG with Pharmacological properties alone)	ATC, category	Recurrent Neural Networks	0.577±0.004	0.401±0.002	0.670±0.003

bioRxiv preprint doi: https://doi.org/10.64898/2026.01.29.702277; this version posted January 30, 2026. The copyright holder for this preprint (which was not certified by peer review) is the author/funder, who has granted bioRxiv a license to display the preprint in perpetuity. It is made available under aCC-BY 4.0 International license.

Supplementary Table 2. Our curated KG is heterogenous, with 4 types of entities and 7 types of undirected edges. Tables below show a breakdown of entities by entity type and relations by entity type. **Supplementary Note 2** provides detailed information about the definition of entities.

Entity pair	Relation types	Number of relations
Drug-Gene	Drug-Target-Gene	16,518
	Drug-Transporter-Gene	3,066
	Drug-Enzyme-Gene	5,241
	Drug-Carrier-Gene	853
Drug-Pathway	Drug-Association-Pathway	3,231
Drug-Category	Drug-Belongs to-Category	599
Drug-ATC	Drug-is_a-ATC	599

Supplementary Table 3. Contingency table used for calculating EOR

	Drugs predicted as DDIs with acalabrutinib	Drugs predicted as non-interacting with acalabrutinib	Total
CYP3 enzyme family	a	b	a+b
All other CYP enzyme families	c	d	c+d
Total	a+c	b+d	a+b+c+d

a = The number of proteins belonging to CYP3 enzyme family across drugs which predicted to have DDIs with acalabrutinib
b = The number of proteins belonging to CYP3 enzyme family across drugs which predicted to be non-interacting with acalabrutinib
c = The number of proteins belonging to all other CYP enzyme families across drugs which predicted to have DDIs with acalabrutinib
d = The number of proteins belonging to all other CYP enzyme families across drugs which predicted to be non-interacting with acalabrutinib

Supplementary Note 1: Choice of graph to represent prior knowledge of the drug entity
We decided to proceed with the iBKH network to represent prior knowledge about the drug entities of interest when predicting co-prescribed drug combinations. The choice was made because iBKH network had the best coverage of the drug molecules we were interested in, and was the most general-purpose for application to future tasks. We studied the impact of varying the components of the curated KG that are used as prior knowledge.
Varying KG components: We first studied the impact of varying the components of the KG used as background knowledge for knowledge injection into the DDI sentence. We found that removing biological process-related entities, such as drug targets and pathways, resulted in the most significant decrease in performance This fits our assumption that drug targets are often critical for DDI prediction. Genes that share similar biological pathways should result in similar predicted DDI risk. While molecular function often overlaps with biological process annotation, biological

bioRxiv preprint doi: https://doi.org/10.64898/2026.01.29.702277; this version posted January 30, 2026. The copyright holder for this preprint (which was not certified by peer review) is the author/funder, who has granted bioRxiv a license to display the preprint in perpetuity. It is made available under aCC-BY 4.0 International license.

process terms are more diverse and represent specific biological end states or outcomes. There was minimal impact on performance when pharmacological properties, such as ATC codes and categories, were removed.

Varying resolution of KG: Given a source drug, we query the KG by identifying its drug targets, transporters, and carrier proteins, with a maximum of $k = 2$ of each node type. These k proteins are then connected to the source drug in the KG. By varying the k parameter, we can study the influence of the density of edges in the graph. We observed that the value of $k = 2$ resulted in the best performance of BEACON. When we reduced the value to $k = 1$, thereby decreasing the density of the graph, we observed that the performance of BEACON decreased. Increasing the value to $k = 3$ or more had minimal impact on performance. We assume that genes that share similar biological pathways should result in similar predicted DDI risk.

Supplementary Note 2.

- *category*: This relation links drug nodes to MeSH (Medical Subject Headings) nodes.
- *ATC*: This relation links drug nodes to ATC (Anatomical Therapeutic Chemical) classification system code nodes.
- *pathway*: This relation links drug or protein nodes to pathway nodes.
- *target*: This relation links drug nodes to protein nodes.
- *enzyme*: This relation links drug nodes to protein nodes that catalyze chemical reactions involving the drug.
- carrier: This relation links drug nodes to protein nodes that are secreted proteins binding to drugs and carrying them to cell transporters.
- *transporter*: This relation links drug nodes to protein nodes representing membrane-bound proteins that shuttle ions into or out of cells.

bioRxiv preprint doi: https://doi.org/10.64898/2026.01.29.702277; this version posted January 30, 2026. The copyright holder for this preprint (which was not certified by peer review) is the author/funder, who has granted bioRxiv a license to display the preprint in perpetuity. It is made available under aCC-BY 4.0 International license.

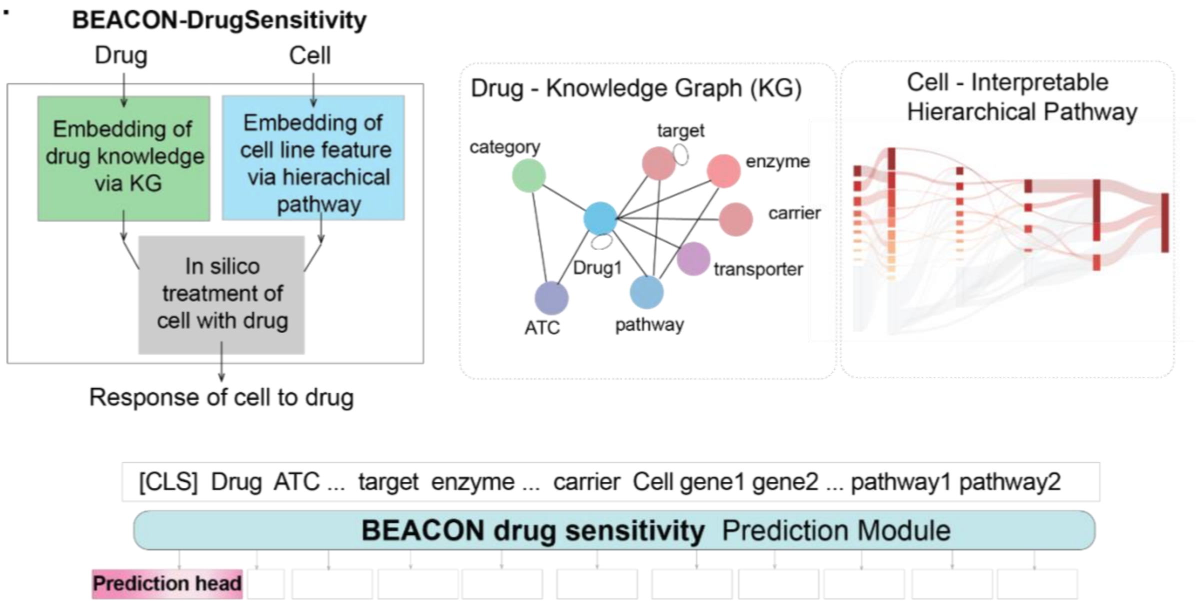

Supplementary Fig. 1: BEACON-DrugSensitivity predicts drug sensitivity by integrating drug embeddings from KGs with cell line embeddings from hierarchical pathways. Each drug is represented via its KG, while each cell line is represented through an interpretable hierarchical pathway structure derived from Reactome. These representations are concatenated as input tokens ([CLS] Drug ATC ... target enzyme ... carrier Cell gene1 gene2 ... pathway1 pathway2) and processed by the BEACON drug sensitivity prediction module to generate response predictions.

bioRxiv preprint doi: https://doi.org/10.64898/2026.01.29.702277; this version posted January 30, 2026. The copyright holder for this preprint (which was not certified by peer review) is the author/funder, who has granted bioRxiv a license to display the preprint in perpetuity. It is made available under aCC-BY 4.0 International license.

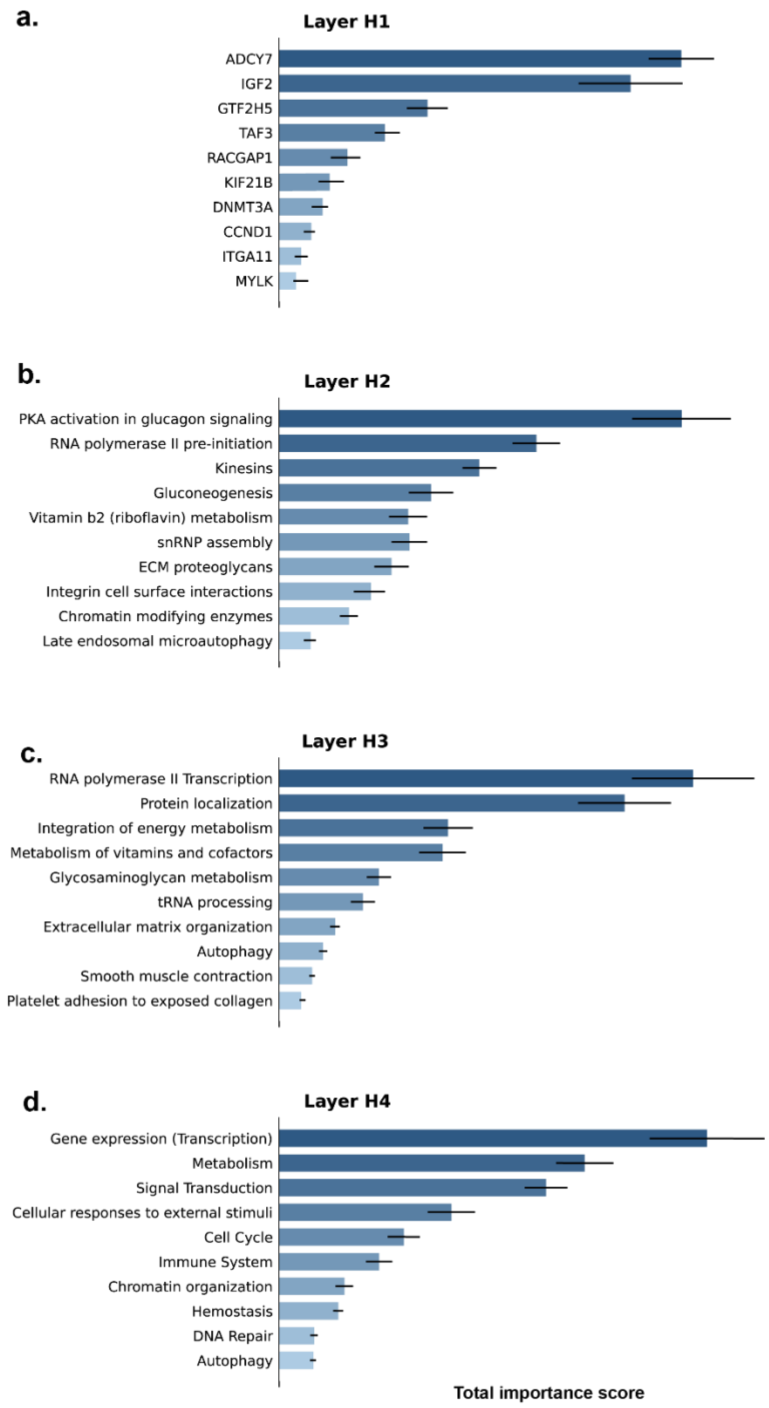

Supplementary Fig. 2: Relative ranking of nodes by total importance score for Trametinib sensitivity prediction. Horizontal bar charts showing the top 10–15 nodes in each layer ranked by total importance score, calculated as the summation of all sample-level importance scores over the testing set. Error bars represent the 95% confidence interval calculated using 1,000 bootstrap cycles.

bioRxiv preprint doi: https://doi.org/10.64898/2026.01.29.702277; this version posted January 30, 2026. The copyright holder for this preprint (which was not certified by peer review) is the author/funder, who has granted bioRxiv a license to display the preprint in perpetuity. It is made available under aCC-BY 4.0 International license.

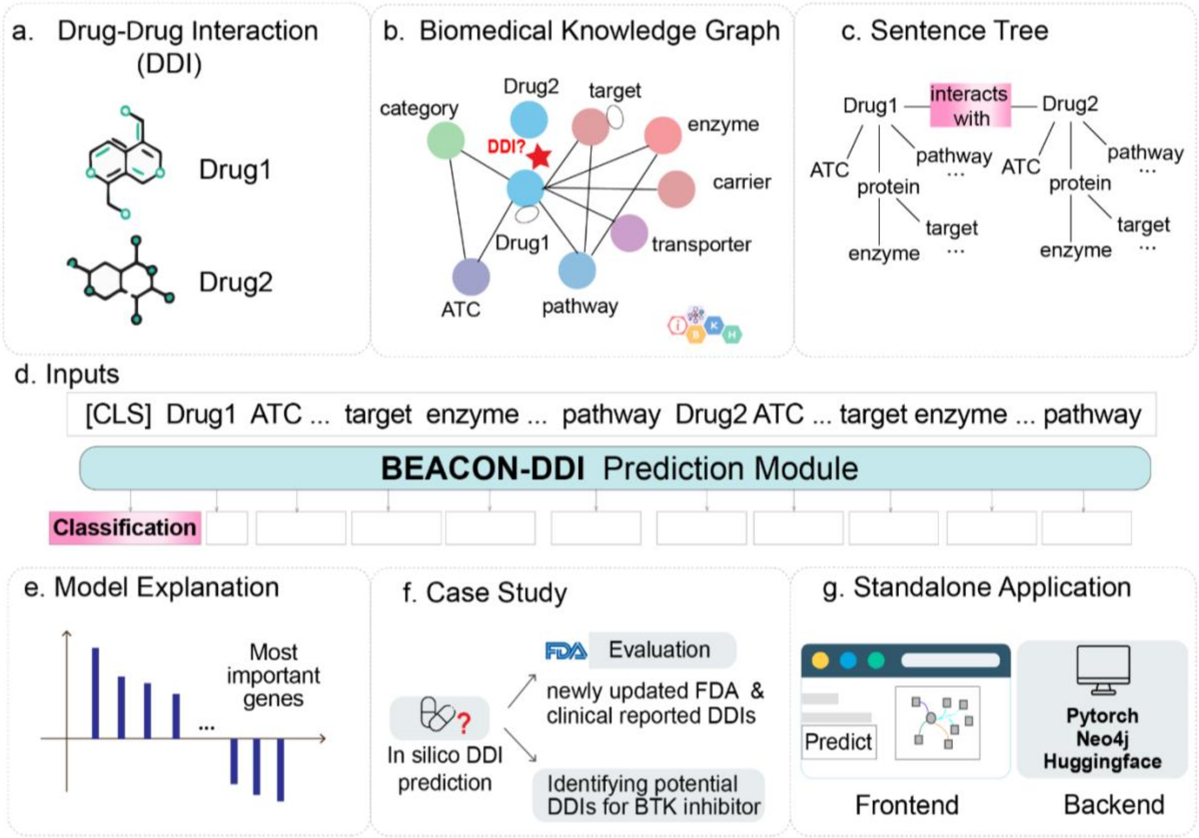

Supplementary Fig. 3: Overview of the BEACON framework on DDI prediction. a. Constructing drug-related multimodal representation through biomedical knowledge integration based on iBKH database. iBKH harmonizes data from 18 publicly available biomedical knowledge sources. We first collected diverse relation data which involves any drug entities in iBKH. Knowledge from diverse biomedical entities was integrated to build our curated drug-centered KG. **b.** We converted each DDI event into a sentence tree via knowledge injection from KG. **c.** The knowledge-enriched tree was translated into natural text and a task-specific prompt was created. The prompt was designed to generate binary class predictions of DDIs. **d.** A transformer-based prediction module was implemented in BEACON for novel DDI predictions. **e.** We explained the language model' reasoning by ranking the most important genes nominated by BEACON-DDI. **f.** We validated our model on a new reference dataset which includes newly updated FDA and clinically reported DDIs. As a proof of concept, we analyzed *in silico* DDI predictions for the BTK inhibitor acalabrutinib. **g.** An interactive, dynamic web server was developed, which allows users to perform on-demand interface predictions using the BEACON-DDI framework.

bioRxiv preprint doi: https://doi.org/10.64898/2026.01.29.702277; this version posted January 30, 2026. The copyright holder for this preprint (which was not certified by peer review) is the author/funder, who has granted bioRxiv a license to display the preprint in perpetuity. It is made available under aCC-BY 4.0 International license.

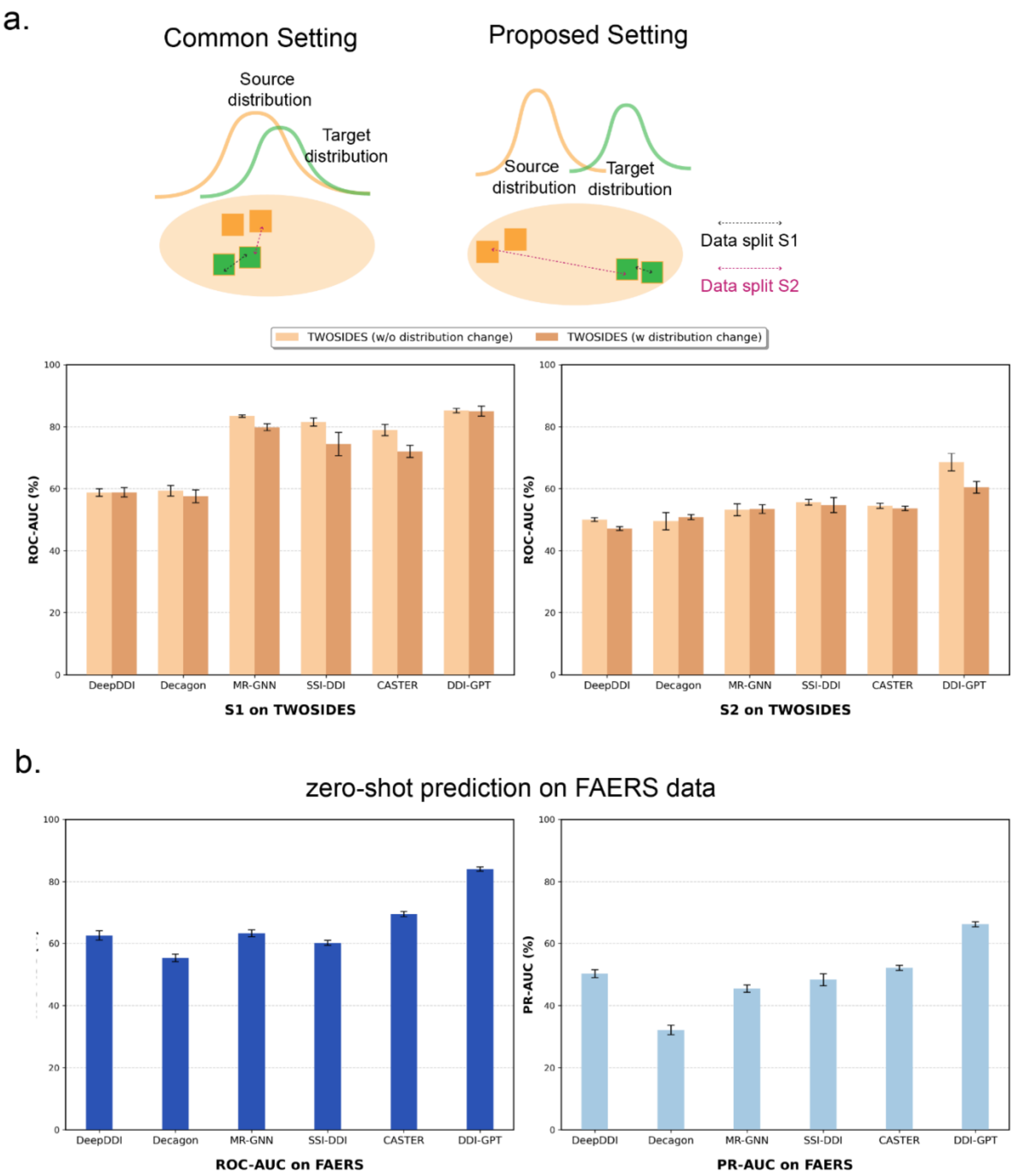

Supplementary Fig. 4: a. A data distribution framework [23] was employed to assess the model performance on generalization of new drug. We benchmarked DDI-GPT with other methods on the TWOSIDES dataset using two data splitting strategies where only one drug was unseen and two drugs were both unseen during the training. **b.** Predictive performances on an independent real-world. FAERS data, assessing the model's zero-shot predictive capabilities.

bioRxiv preprint doi: https://doi.org/10.64898/2026.01.29.702277; this version posted January 30, 2026. The copyright holder for this preprint (which was not certified by peer review) is the author/funder, who has granted bioRxiv a license to display the preprint in perpetuity. It is made available under aCC-BY 4.0 International license.

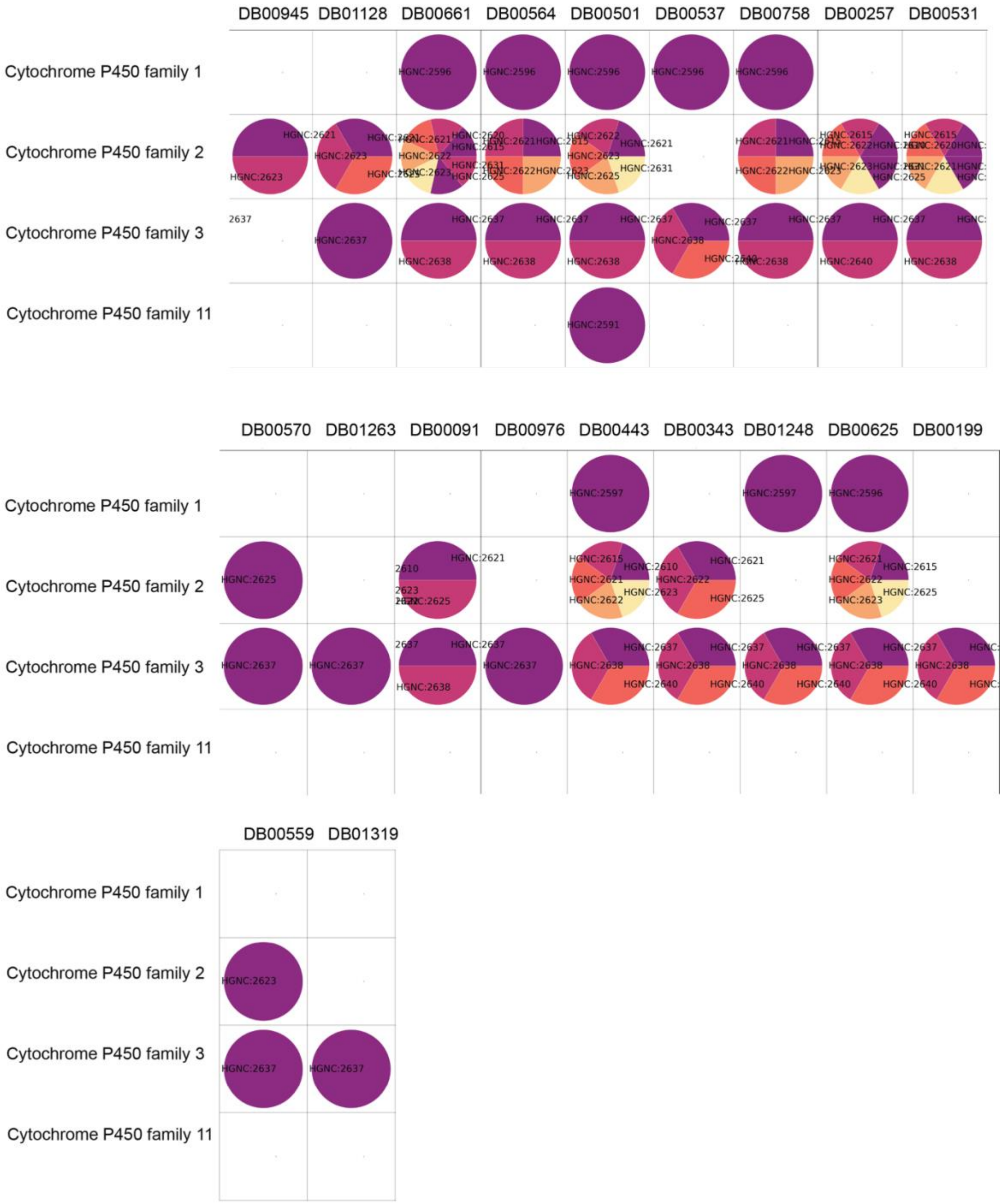

Supplementary Fig. 5: Enzyme families of acalabrutinib predicted DDI drug for disproportional analysis in **Supplementary Table 3**. The columns are drugs standardized with DrugBankID, and the rows are the CYP enzyme families. Each circle indicates a drug target that belongs to a certain family, presented by HGNC ID. If empty, this means that the drug is absent from targets metabolized by that enzyme family.

bioRxiv preprint doi: https://doi.org/10.64898/2026.01.29.702277; this version posted January 30, 2026. The copyright holder for this preprint (which was not certified by peer review) is the author/funder, who has granted bioRxiv a license to display the preprint in perpetuity. It is made available under aCC-BY 4.0 International license.

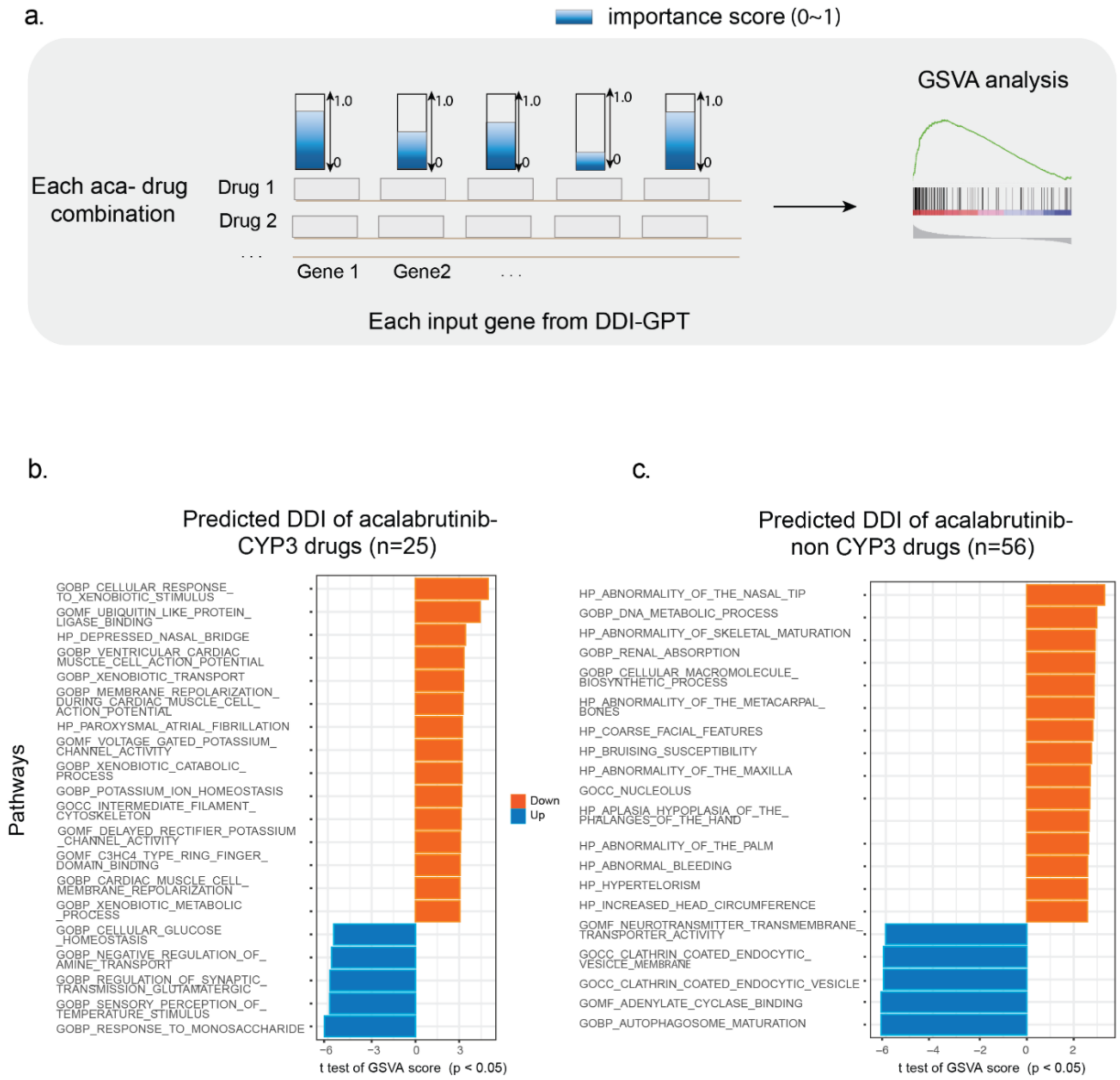

Supplementary Fig. 6: **a.** BEACON-DDI evaluation module uses importance scores to estimate the contribution of the word to the prediction, which can be computed efficiently by using perturbation-based approximation. The idea is to perturb the contribution of the word by adding a Gaussian noise and measure the magnitude of change in the prediction score. **b.** Genes are clustered based on their importance value by an unsupervised K-means clustering. Pathway analysis is performed on each cluster.

bioRxiv preprint doi: https://doi.org/10.64898/2026.01.29.702277; this version posted January 30, 2026. The copyright holder for this preprint (which was not certified by peer review) is the author/funder, who has granted bioRxiv a license to display the preprint in perpetuity. It is made available under aCC-BY 4.0 International license.

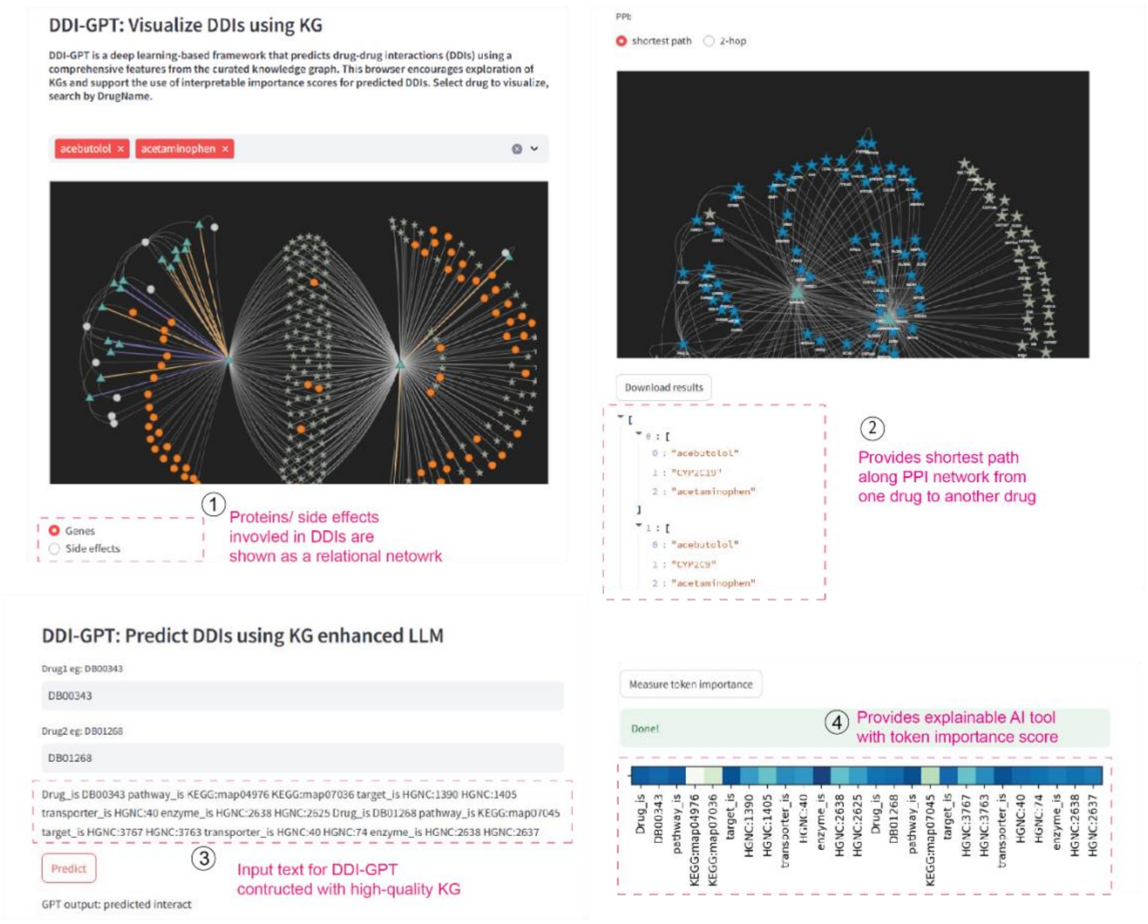

Supplementary Fig. 7: BEACON web server for knowledge-enhanced DDI exploration**.** The user submits a pair of drugs, referenced by DrugBank ID, and the input sentence is generated by incorporating drug-related biomedical entities from KG. The input is then set to a cluster running the prediction pipeline, where it is processed. Once the interface prediction is complete, the BEACON web server will be updated to visualize important words generated by the explanation module. The BEACON web server allows users to explore the KGs that were used by the BEACON framework, as well as various drug-protein-drug interaction networks and drug-side-effects-drug interaction networks. The user can view the shortest paths or two-hop paths between combinations of drugs.
